# Association between severe obesity and graft outcomes in pediatric heart transplants

**DOI:** 10.1016/j.xjon.2025.07.005

**Published:** 2025-07-18

**Authors:** Laksha Lohano, Prem Singh, Arsalan Hyder, Niksha Giyan, FNU Venjhraj

**Affiliations:** a Liaquat University of Medical and Health Sciences Jamshoro, Jamshoro, Pakistan; b Department of Emergency Medicine, Dow University of Health Sciences, Karachi, Pakistan; c Department of Internal Medicine, Dow University of Health Sciences, Karachi, Pakistan; d Department of Internal Medicine, Shaheed Mohtarma Benazir Bhutto Medical College Lyari, Karachi, Pakistan

To the Editor:

**Figure:**
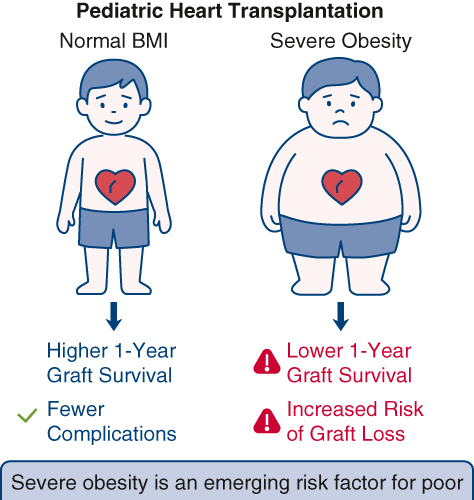

We recently reviewed the article by Sparks and colleagues,^1^ which found lower long-term survival rates in children with extreme obesity getting heart transplants. We applaud the authors in conducting this thorough investigation. Nevertheless, we think a few additions that could improve the study's validity are worth considering further.

Even without extracorporeal membrane oxygenation support, severe obesity remained a risk factor for graft loss or death, as evidenced by the fact that more than half of the extremely obese patients passed away by the 10th year. It is important to note that, despite the fact that body mass index is a widely used screening tool for obesity in health care and public health practice, current research supports that it is suboptimal, at least for children younger than age 9 years. Body mass index *z* scores serve as a weak-to-moderate indicator of the amount of fat in a body and of overall fat mass in young children. This signifies that more precise indicators of adiposity are required during early infancy for better treatment.^2^

Secondly, patients ages 2 to 8 years were excluded from the study, which reduces its external validity and creates a significant knowledge gap. Thus, it is infeasible to determine how anthropometric measurements and obesity influence transplant outcomes for patients in this age group. Because newborn infants’ risk variables differ from those of other pediatric patients, this could influence bedside decision making. To predict these age-related changes, more studies should be conducted.^3^

Furthermore, hormone therapy provided to donors has been linked with improved outcomes. Donors classified as marginal donors—such as those with head injury, high-dose inotropes, or those receiving cardiopulmonary resuscitation—do not adversely influence survival for pediatric heart transplantation. A lower weight ratio does not detrimentally influence the outcome; however, a lower donor body surface area is associated with poor posttransplant outcome. A negative correlation between ischemia time and 1-year survival is reported.^1^ Donor variables have little effect on survival for infants younger than age 6 months, but longer ischemia time and wider donor-to-recipient age difference escalate early mortality for children older than age 10 years.^4^

Lastly, children who are severely obese at the time of heart transplantation are more likely to experience graft loss and coronary allograft vasculopathy (CAV), the chief cause of late graft failure, particularly 3 years posttransplant. Despite limited evidence, greater long-term risk of death among obese patients indicates faster CAV progression. Early development of CAV is more difficult to treat because it frequently goes undiagnosed and advances silently. Following a transplant, obesity is closely associated with the development of CAV, as are other cardiovascular risk factors like diabetes and high cholesterol level.^5^

## Conflict of Interest Statement

The authors reported no conflicts of interest.

The *Journal* policy requires editors and reviewers to disclose conflicts of interest and to decline handling or reviewing manuscripts for which they may have a conflict of interest. The editors and reviewers of this article have no conflicts of interest.
